# Understanding disability from a secondary data lens perspective: Evidence from consultations with members of the public with disabilities in the UK

**DOI:** 10.1371/journal.pone.0318409

**Published:** 2025-02-21

**Authors:** Eirini-Christina Saloniki, William Lammons, Sarah Markham

**Affiliations:** 1 Department of Primary Care and Population Health, University College London, London, United Kingdom; 2 NIHR Applied Research Collaboration North Thames, London, United Kingdom; 3 Department of Biostatistics and Health Informatics, King’s College London, London, United Kingdom; University of the Witwatersrand Johannesburg, SOUTH AFRICA

## Abstract

Disability is a multifaceted phenomenon, which complicates data collection about people with disabilities in surveys and censuses. A central issue is that the multiple underlying theoretical models about disability are seldomly made explicit yet strongly determine how data are collected and analysed by governments and organisations. It is crucial that such models together with other information about disability and its measurement are accessible and understood by everyone. This study comprised several UK survey searches for disability or disability-related questions and a series of consultations with members of the public with lived experience of disability to understand their perceptions of theoretical models of disability in survey questions. The findings highlighted the importance of continued involvement of people with lived experience in technical research activities. They further revealed that members of the public with lived experience can effectively become familiar with theoretical models of disability and how to analyse them in relation to survey questions subject to careful preparation, including practical examples.

## 1. Introduction

### 1.1. Disability: a complex phenomenon

Almost everyone will experience some form of disability during their life [1]. In the UK, 16 million people live with a disability, and this is likely to increase due to a rise in chronic and comorbid health conditions and population ageing, among other factors [2]. Disability has been at the forefront of recent governmental strategies (for example, National Disability Strategy in the UK), highlighting that research in this sphere is necessary, vital and timely.

Disability is a complex and multifaceted phenomenon; its definition varies with context, purpose and data, which complicates data collection about people with disabilities in surveys and censuses [3]. A key issue is that there are several underlying theoretical (otherwise known as conceptual) models about disability (e.g., medical model, social model, biopsychosocial model) that are rarely made explicit, yet drive how data are collected and analysed by governments and organisations. Also, anti-discrimination legislation (e.g., Equality Act 2010 in the UK) further contributes to characterising disability. All these issues leave little space for a gold standard disability definition. While views supporting an integrated disability definition exist, it is recognised that disability is an ‘evolving concept’, making the adoption of a common definition more challenging [4,5]. Despite the efforts to ‘harmonise’ disability questions across UK surveys [6], data collection on disability, particularly around the wording of the questions, remains inconsistent across surveys.

At the same time, when searching for disability or disability-related questions in surveys (e.g., governmental, national, cross-national) and censuses, it is often difficult to identify if they exist, where they are, and in what form, as they are scattered across several online files, catalogues and URLs. Searching and cross-referencing multiple surveys and databases in this context is not only time-consuming, but also requires prior research experience, familiarity with jargon and technical skills such as navigating disparate domains in different formats. As a result, the searches can be difficult to conceptualise, navigate and importantly are less accessible by people with disabilities and their advocacy groups. To reduce complexity while engaging more researchers, policymakers and service users (with disabilities or not) in this area of research, it is imperative that information on the concept of disability and its measurement in surveys is identified and understood by everyone, especially people with disabilities.

To facilitate this process, this study’s contribution is twofold: (a) it identified all disability and disability-related questions from the largest UK repository for secondary survey data and, (b) conducted a set of patient and public involvement and engagement (PPIE) consultations to gain some understanding of public perceptions of theoretical models of disability in survey questions.

### 1.2. PPIE with people with disabilities

People with disabilities are often excluded from research and involvement activities [7,8] or tokenised while participating in involvement [9]. Recent articles have called for ‘empowerment’ of those being involved or involving them so that they participate in the creation of research and knowledge related to disability [7,8]. In other words, consulting and working with members of the public on various aspects of the research, including more technical elements less accessible to a public audience. The National Institute of Health and Care Research (NIHR) defines ‘consultation’ as one such modality of PPIE; ‘when you ask members of the public for their views and use these views to inform your decision making’, and consultation can focus on ‘any aspect of the research process’ [10]. In line with the NIHR guidelines, and considerate of the limited opportunities for PPIE activities for people with disabilities, we conducted PPIE consultations with members of the public with disabilities who were keen to discuss and provide their views on research related to disability and then apply these views to map survey questions to theoretical models of disability [11–13].

The study aimed to: (a) facilitate researchers, policy makers and the public in understanding the multifaceted concept of disability identified in UK surveys available for secondary use and, (b) reduce widespread waste of disability/disability-related questions and the corresponding insights they offer in relation to different theoretical models of disability through the eyes of members of the public with lived experience of disability.

## 2. Methods

### 2.1. Survey searches

We identified all disability and disability-related questions from surveys in the largest UK repository for quantitative and qualitative social science and humanities research data, namely the UK Data Service. ECS was a registered user of the repository which provided her with access to non-sensitive information about the different surveys (e.g., survey description, questionnaires) via an End User Licence Agreement. Such information falls under research exempt from ethical approval according to the authors’ institution. The search of the relevant questions involved three steps: (a) development of search terms to identify eligible surveys, (b) screening of questionnaires in eligible surveys to identify questions associated with disability, (c) extraction of the relevant questions and grouping into different sets in preparation for the consultations with the members of the public with lived experience. The search terms considered were: [impair*] [disab*] and [long-term ill * OR condition * OR disab * OR impair*], the time reference was 2010-2023 (to avoid changes made prior to the latest UK anti-discrimination legislation) and the country of interest was the UK.

### 2.2. Members of the public and recruitment

ECS and WL developed an invitation for participation which was reviewed by the Virtual Document Review Panel (VDRP) within Applied Research Collaboration (ARC) North Thames – a panel of public collaborators who regularly review materials related to research projects [14] – and subsequently amended to address the panel’s comments and ensure accessibility. The invitation included a lay synopsis of the study, an outline of the lived experience characteristics required for the study and types of activities to be involved, compensation details for their involvement, funder information and key researchers’ contact details. According to the NIHR [13], consultations with members of the public are classed as involvement (i.e., collecting opinions rather than study data), and thus do not require ethical approval. The invitation was circulated via the NIHR’s People in Research platform which lists opportunities for public involvement in NHS, public health and social care research [15]. Additional dissemination channels included an email to all ARCs’ PPIE teams, social media and newsletters of the organisations involved as well as other contacts and their networks in charitable organisations, such as Scope and Shaping our Lives [16,17]. The invitation remained open between 12 December 2022 and 31 January 2023. Members of the public who identified as having a disability and resided in the North Thames area (i.e., North East London, North Central London, East London, and Mid and South Essex) were invited to respond to the invitation with their interest by return email.

The organisers of the PPIE consultations (ECS and WL) received a total of 22 expressions of interest, of which 10 were from the North Thames area, and the remaining 12 were from the rest of the UK. These were assessed on a case-by-case basis in two stages: (a) responses by return email to the question: ‘Do you have any experience with data and survey focused public involvement work?’, and (b) a 20-30-minute one-to-one call with ECS or WL to learn more about their experiences and interests. Each consultation participant had the opportunity to notify ECS and WL of any reasonable adjustments they required which were also taken into consideration (for example, consultations not to take place in the mornings, regular comfort breaks). ECS and WL reviewed all responses, consolidated notes and together with two members of the public with lived experience of disability (SM and AC; known to ECS and WL through ARC NT PPIE activities) who acted as advisors, assembled a convenience sample based on respondents’ stated interest and advisors’ feedback. We aimed for an inclusive and diverse representation of disability to optimise coverage while engaging those typically excluded from research. In this regard, given the low interest from within the North Thames area and the high calibre of respondents from outside North Thames, all expressions of interest were considered irrespective of the geographical area they represented. All respondents (successful or not) were notified of the decision via email or a telephone call, based on their stated preference. A total of seven respondents were invited to participate as they reflected a variety of lived experiences and interests for the study. One of them – known to WL from other PPIE activities – abstained prior to the first session for personal reasons. As per NIHR guidelines, participants were remunerated at £25/hour and another £5/session to support the cost of internet [18]. ECS and WL knew none of the final 6 participants from other PPIE activities. Further communications with the participants were conducted through email or a telephone call based on their preference. ECS and WL explained in detail to each participant what sessions would consist of in one-to-one meetings before the actual sessions took place to assure that all participants adequately understood everything involved and were able to consent. Public contributors consented to participating in sessions verbally. They also consented to recordings verbally, and the number of recordings were minimised overall. Additionally, regular ongoing checks were conducted with everyone to support access needs and assure they continued to consent, with the two advisors present in this process.

### 2.3. PPIE consultation structure

We used public involvement methodologies to explore understandings, experiences and perceptions of theoretical models of disability in survey questions amongst members of the public. A total of four consultations were conducted with all six participants. The topics and materials for each session had been communicated ahead through email. ECS and WL co-facilitated the sessions with two note-taking facilitators (SM and AC) present in all four sessions. All sessions were held virtually through Zoom to best logistically support consultation participants’ requirements and participation from across England. Two sessions were recorded after receiving consent from all participants at the start of each session. The remaining sessions involved introduction and training of the members of the public and gathering overall study feedback and were not recorded but notes were taken. We ensured that all had the greatest opportunity to join the sessions and participate by sending a reminder, prompting and encouraging chat communications, reading any posted messages aloud, allowing participants to deactivate video if they did not feel comfortable and incorporating up to two comfort breaks in each session.

The consultations were structured as follows: 1. Brief introduction and explanation of ground rules (5 minutes); 2. Presentation by ECS introducing and explaining important concepts or techniques (20-30 minutes depending on the session); 3. Collective group discussion with all members of the public (20 minutes); 4. Comfort breaks (up to 10 minutes each). The topics of each consultation were: 1. Introduction to the study: become familiar with the key concepts and theoretical models of disability; 2. Mapping exercise: collaboratively ‘map’ different survey questions to theoretical models of disability; 3. Outputs: discuss how best to utilise the findings from the mapping exercise and how to relay the information in a concise, accessible and lay way; 4. Feedback: share the experience and learnings from this consultation to help design future studies in this area. For the purpose of this paper we will focus on the first and second session. A reflective piece on the remaining sessions and the overall consultation process is available elsewhere [19]. Participants 6 and 3 did not attend the first and second session respectively for personal reasons but they were briefed about the content of the session they missed in the following session. The second session was recorded with Zoom’s features and used to create a raw transcript that ECS edited, cleaned and pseudonymised. ECS and WL reviewed the transcript, discussed discrepancies and WL made clarifying edits. Notes from the two note-takers were collated and incorporated in the analysis.

### 2.4. Training the members of the public

We assumed that the members of the public did not have any prior knowledge about disability-affiliated concepts, nor were they familiar with any of the theoretical models that have been extensively used to conceptualise disability. The first consultation was an opportunity for them to get to know each other while also familiarise themselves with the different concepts and ensure that they could ask questions early on. This session was not only paramount for the mapping exercise planned for the second consultation but also acted as a refresher of the basic PPIE principles, emphasising how such activities are crucial for the proposed study. Participants were introduced to the concepts of: impairment, functional limitation, activity limitation and participation restriction, in this order, using a theoretical definition followed by practical examples. These concepts were crucial for subsequently describing the three most prominent theoretical models of disability, namely the medical model, social model and biopsychosocial model [20,21].

### 2.5. Mapping exercise structure

Each set of questions identified through the survey searches was presented separately to the public contributors in the second consultation. They were asked to choose the theoretical model from the three they learned about in the first session – in the following order: medical, social, biopsychosocial – that best reflected these questions. If they felt that none of the existing models was suitable, they could indicate so. In cases where sets of questions had been previously used as a pair, it was made explicit to consider them as a pair when choosing the appropriate model. This type of exercise closely resembles the process of concept mapping whereby (a) key concepts are being identified, (b) a rank order of these concepts from the broadest to the most specific (order can be approximate and reversed) is established, (c) a map is created and (d) close links between the different domains of the map are identified [22].

## 3. Results

### 3.1. Public contributors

The group of members of the public with disabilities was not intended to be representative of the general population but rather a subset interested in consulting on research. Their perspectives were based on their own lived experience as someone with disability. We did not purposely collect standard demographic data in order to give them the opportunity to speak about their identities, backgrounds and opinions as and when they felt appropriate. They however, had prior PPIE experience with quantitative survey data, and some of them had a mix of qualitative and quantitative experience in PPIE activities. They all identified as having a disability, including physical (e.g., amputee, glaucoma, fibromyalgia), mental (e.g., bipolar), neurodiverse (e.g., ADHD, autism), and resided in different areas (East London, North London, Hertfordshire, Leicester, West Yorkshire) across England.

### 3.2. Survey questions

The searches in the UK Data Service brought about 72 survey series which, after excluding duplicates, were reduced to 44 surveys. Questionnaires were manually searched in each survey between 2010 and 2023 for disability or disability-related questions that have been frequently used in the literature to determine disability, including disability benefits, but the latter were not considered in the mapping exercise nor were they counted in the shortlisted questions. Of these, seven surveys did not have any relevant questions, four surveys did not have accessible questionnaires with End User Licence Agreement and one survey did not collect data for the years of interest (i.e., 2010 onwards). As a result, a total of 32 UK surveys were considered in the final sample (see S1 Table for the full list). The surveys yielded a total of 137 disability or disability-related questions, with four questions on average per survey for the years of interest. The wording of the questions varied, in part reflecting the gradual legislation implementation, and the questions were direct or indirect. Examples of indirect questions included: ‘Do you have a long-standing physical or mental impairment, illness, or disability that you expect will last for at least 12 months?’, ‘Does this long standing illness or disability affect the kind of paid work that you might do?’ and direct: ‘Can I just check, do you consider yourself disabled?’. To reduce the burden of the mapping exercise on participants, ECS and WL discussed how to best group the questions in a meaningful way, acknowledging that there was overlap across the different surveys and some questions had been previously paired together to determine disability. Following this discussion, four main sets of questions were identified (see Table 1), two of which had been extensively used in social research to define disability [23]. The first three sets had been used in pairs whereas the fourth set included questions that had been used separately to identify disability. In this latter set, the questions directly require respondents to identify themselves as having a disability and/or indicate if another member inside or outside the household has a disability. Small wording amendments to the questions in each set across the different surveys were also highlighted but were not the focus of the mapping exercise (see notes in Table 1).

**Table 1:** Groups of questions selected for mapping exercise.

**Set A (consider as a group)**
1. Do you have any long-standing physical or mental impairment, illness or disability? By ‘long-standing’ I mean anything that has affected you over a period of at least 12 months or that is likely to affect you over a period of at least 12 months.
2. Does this/Do these health problem(s) or disability(ies) mean that you have substantial difficulties with any of these areas of your life? Mobility (moving around at home and walking) Lifting, carrying or moving objects Manual dexterity (using your hands to carry out everyday tasks) Continence (bladder and bowel control) Hearing (apart from using a standard hearing aid) Sight (apart from wearing standard glasses) Communication or speech problems Memory or ability to concentrate, learn or understand Recognising when you are in physical danger Your physical co-ordination (e.g., balance) Difficulties with own personal care (e.g., getting dressed, taking a bath or shower) Other health problem or disability None of these
**Set B (consider as a group)**
1. Do you have any physical or mental health conditions or illnesses lasting or expected to last for 12 months or more?
2. Does your condition or illness/do any of your conditions or illnesses reduce your ability to carry-out day-to-day activities?
**Set C (consider as a group)**
1. Do you have any health problems or disabilities that you expect will last for more than a year?
2. Does this health problem affect the kind of paid work that you might do?
3. Does this health problem affect the amount of paid work that you might do?
**Set D (consider separately)**
1. Can I just check, do you consider yourself disabled?
2. Are you/they registered as a disabled person (or as visually impaired) with the local council/social services?
3. May I check, is there anyone living with you who is sick, disabled or elderly whom you look after or give special help to, other than in a professional capacity (for example, a sick or disabled (or elderly) relative/husband/wife/child/friend/parent, etc)?

Notes: Wording changes in each set are bolded. Set A (alternative wording): 1. Do you have any long-standing illness, disability or **infirmity** – by ‘long-standing’ I mean anything that has troubled you over a period **of time** or that is likely to affect you over a period **of time**? 2. Does this illness or disability (Do any of these illnesses or disabilities) **limit your activities in any way**?. Set B (alternative wording): 1. Do you have any illness **or disability** that you expect will last for at least 12 months? 2. Does this condition limit your ability to carry-out **normal** day-to-day activities **in any way**? **[Please consider whether you are affected while receiving any treatment, taking medication, or using any devices, such as hearing aid.]**. Set C (alternative wording): 1. Could I just check, do you have any **long-standing illness, health problem or disability that limits your daily activities or the kind of work that you can do**? 2. Do you have any health problem that **limits the kind or amount of paid work you could do, should you want to**?.

### 3.3. ‘Concept mapping’

The first pair of questions received almost full consensus with four votes on the biopsychosocial model. The final session participant was unable to map the questions to one theoretical model as a pair.

*‘So I think it fits the third one because the first question is to do with physical and mental disability which is to do medical and social as well… And the second question, … it would mean that I we have medical problems as well health problems which is medical, and it also comes then it goes on to become a social problem as well which is why I think, the third model you know, relates to those two questions.’* [Participant 6]

*‘… the biopsychosocial I think it gives them power to the individual as well to be able to have some control and some say in their disability and impairment. But also that includes that it is not about me having to change. It is about society having to change.’* [Participant 5]

The second set of questions was more inconclusive, with two participants unable to map the questions as a pair to one theoretical model and one participant not able to decide. One participant voted in favour of the biopsychosocial model.

*‘Reading them, I think the medical, the wording in there where it says consequences of physical or mental restriction, disability as the consequence of physical and mental restriction that ties in on the social. You have got disability or something a person experiences which ties in. But then again, I think bio combines the two. So to me the bio seems to fit better…’* [Participant 2]

Another participant mapped this second set onto the medical model.

*‘It seems almost like the medical model. And the reason why I am saying that is because, as you know, when you claim one of the things about the work, capability, assessment and this is the way that they ask some of the questions on there. And it is more it does not really look at what things like social it takes into account, or anything like biopsychosocial. It just specifically looks at your condition as a medical condition and how that affects you doing certain things. So when I look at this, it just reminds me of how the way these questions are phrased in relation to those assessments, and it almost seems like a medical model really.’* [Participant 5]

The third set of questions generated a lot of discussion around the wording of the questions. Participants generally found it difficult to map the questions (as a set) onto one theoretical model of disability and there was no consensus.

*‘Yes, so I actually think that the last two questions focus more fit to the biopsychosocial model. And the first one, it is seeming like the medical. I was saying that the second and third one they seem like the biopsychosocial for me but the first one I am trying to link it to the medical model, but it is not fit. And because from a medical perspective it is more about the condition, the care and results of the care. So and then this question is assuming, like, how your perception of the disability and I want to fit to the social model. But there then, again, there is no element of social interaction in the question… really, the second one, third one they fit the biopsychosocial model for me but the first one it is sticky.’* [Participant 4]

The fourth set of questions, albeit it was made explicit that they should be mapped separately, gathered very different views with no consensus amongst the six participants. One participant, although invited to express their view, did not provide any insight on the mapping of any of the questions in this last set. Another only shared their view about one of three questions. The question ‘Can I just check, do you consider yourself disabled?’ was perceived by two participants as closest to the biopsychosocial model.

*‘… the first one as bio because I think it is a combination of things.’* [Participant 2]

The question ‘Are you/they registered as a disabled person (or as visually impaired) with the local council/social services?’ was seen by one participant as closest to the medical model,

*‘So the second question that seems to almost be like that it is in with a medical model because it is to get a service or to get accessibility to services or a particular organization. Well, it seems almost that you have got to show proof of your disability. It seems to align with the medical model.’* [Participant 5]

and another considered this question more aligned to the social model.

*‘The second one I would say social because it is more down to the way we are seen by social services and things like that.’* [Participant 2]

The final question ‘May I check, is there anyone living with you who is sick, disabled or elderly whom you look after or give special help to, other than in a professional capacity (for example, a sick or disabled (or elderly) relative/husband/wife/child/friend/parent, etc)?’ was mapped by one participant onto the social model and another onto the biopsychosocial model.

*‘... three as social because they to me come under like social care and the way they’re treated by social services, or on the how you would get from that kind of area…’* [Participant 2]

## 4. Discussion

### 4.1. Main findings

We used an activity typically reserved for technical research practice – mapping survey questions to theoretical models – and conducted this with members of the public who have lived experience of disability. Overall, the activity produced diverse connections between question groups and theoretical models of disability, possibly influenced by how disability is conceptualised within each model.

It is worth noting that in the medical model, disability is viewed as a ‘medical phenomenon that results in limited functioning that is seen as deficient’ [24]. In that sense, any limited functioning linked to an impairment is inherently disabling to the individual, and not linked to society. By contrast, in the social model, impairment is separated from disability – impairment is ‘perceived as an abnormality of the body’ while disability is something an individual experiences and ‘any disadvantages are caused by society’ [24]. By doing so, the social model, and subsequently the biopsychosocial model, continue to relegate the disabled body to medicine [25]. The biopsychosocial model aims at bringing the medical and social model together by taking a broader approach to disability where a person’s level of functioning is a dynamic interaction between health conditions and contextual factors, the latter including personal and environmental factors [26–28].

Collectively, among the members of the public there seemed to be a slight preference for the biopsychosocial model, likely because this model resonated most closely with their lived experiences and understanding of disability as a dynamic and multifaceted concept that includes medical, psychosocial, and additional elements. As we moved through different question groups, consultation participants’ responses often splintered between a mix of preferences for the biopsychosocial model, some preference for the medical model, and indecision for one particular model. Participants frequently felt a need to separate the individual questions within one question group, and map these questions to models individually, as opposed to mapping the entire group, where needed. This likely made it easier and broke down the mapping process into individual and more manageable pieces while also allowing participants to focus on the unique nuances of each aspect of the questions. Further, they, at times, critiqued the survey questions themselves, finding shortfalls or inadequacies in their wording or explanations of disability, and analysing them in different ways that were relevant to the theoretical models or surveys, such as considering whether the question was qualitative or quantitative.

All of this reveals two significant findings. First, people with lived experience of disability understand disability as a complex, multifaceted phenomenon, and that adequately understanding and measuring it through surveys is equally complex and difficult. Second, despite the diverse opinions during the mapping exercise, members of the public with lived experience succeeded in understanding what theoretical models of disability are and how to analyse them in relation to survey questions.

### 4.2. How does our exercise relate to existing mapping evidence?

To our knowledge, no explicit mapping of survey questions to theoretical models of disability nor public contributors’ insights of such a link are evident in the UK literature, but it is understood that the social model has dominated most disability studies in the UK [29]. In parallel, there is scarce reference in UK survey questionnaires or technical guides to certain theoretical models of disability. For example, the questions from the second group set, directly referring to impairment and health conditions, have been linked to the medical model, as in these questions people with disabilities are ‘defined by their impairment or health condition, which is perceived as causing dependence and a need for treatment or care’ [30]. One participant in the study also associated this group set to the medical model. One could argue that given the similarities in wording in the first group set and the direct connotation of health conditions, this set is also linked to the medical model. However, there are questions that have been linked to models other than the medical – specifically, one of the questions in the fourth set (i.e., ‘Do you consider yourself disabled?’) has been linked by some to the social model as the individual ‘might consider themselves to be disabled by virtue of society disabling them as opposed to their medical condition causing them to be disabled’ while by others it resembles an identity model [31–34]. The same could be said for the rest of the direct questions in the fourth group set. The association of the fourth set with the social model was not shared with the participants’ choice of the biopsychosocial model. Recent recommendations by the UK’s Inclusive Data Taskforce emphasised that lead organisations in disability data collection (e.g., Office for National Statistics) should transition all disability measures to approaches that are more closely affiliated with the social and biopsychosocial approaches [35].

### 4.3. What could have been done differently in the mapping exercise?

Our experience with the mapping exercise has provided clarity on how such exercises can be conducted more effectively in the future. One aspect to consider is the inclusion of an actual voting mechanism into the exercise. This could serve as an interesting contrast to the indecision and variation in responses seen during the activity. Additionally, further training on what mapping generally entails and how it is done in practice would likely be beneficial to members of the public with lived experience in mapping processes. In this regard, ‘practice’ exercises can be included to address natural hiccups in the process, such as indecision, inability or unwillingness to participate and variation in voting.

## 5. Conclusion

This study showcased how mapping survey questions to theoretical models of disability can be conducted with members of the public who have lived experience of disability. Despite some difficulties encountered in completing the activity, which could be due to the fact that models of disability used to inform survey questions are not fully representative of the experiences of people with disabilities, it produced varying connections between question groups and theoretical models of disability.

Future research activities should further continue the learnings from this study – to conduct technical research activities by preparing members of the public with lived experience and doing the research exercises with them, especially for mapping surveys to theoretical models. They should, however, do so while considering how much preparation is actually needed in order to effectively map questions to the theoretical models.

People with lived experience of disability can provide essential insights into how to more completely understand key concepts that drive research, and when adequately given a platform to learn about it and engage with the ideas in an inclusive environment, can serve as close participants in otherwise technical research activities and exercises.

## Supporting information

S1 Table Disability or disability-related questions in UK surveys. (DOCX)
