# Supplementary material for: Understanding disability from a secondary data lens perspective: Evidence from consultations with members of the public with disabilities in the UK
Source: PLoS One. 2025 Feb 21;20(2):e0318409. doi: 10.1371/journal.pone.0318409 (PMC11844830; doi:10.1371/journal.pone.0318409)
Supplement: S1 Table — (DOCX) [file pone.0318409.s001.docx]

**Supplementary material**

Table S1. Disability or disability-related questions in UK surveys

| **Survey^†^** | **Disability or disability-related questions^*^** | **Year** | **Wave** | **Total no. of different questions** |
| --- | --- | --- | --- | --- |
| Active Lives and Active People surveys (Sport England) | - Do you have a long-standing illness, disability or infirmity? By longstanding I mean anything that has troubled you over a long period of time or that is likely to affect you over a period of time.  - Does this illness or disability limit your activities in any way? | 2010-16 | NA | 5 |
|  | - Do you have any physical or mental health conditions or illnesses that have lasted or are expected to last 12 months or more? - Do these physical or mental health conditions or illnesses have a substantial effect on your ability to do normal daily activities? | 2016-21 | NA |  |
|  | - Does this disability or illness affect you in any of the following areas? | 2010-21 | NA |  |
| Annual Population Survey/Labour Force Survey | - Do you have any health problems or disabilities that you expect will last for more than a year? | Jan-Dec 2010-13 | NA | 9 |
|  | - Does this health problem affect the kind of paid work that you might do? - Does this health problem affect the amount of paid work that you might do? - Which of these is your main health problem/disability? - Do these health problems or disabilities, when taken singly or together, substantially limit your ability to carry out normal day to day activities? If you are receiving medication or treatment, please consider what the situation would be without the medication or treatment? | Jan-Dec 2010-21 | NA |  |
|  | - Do these health problems or disabilities, when taken singly or together, substantially limit your ability to carry out normal day to day activities? If you are receiving medication or treatment, please consider what the situation would be without the medication or treatment? | Jan-Dec 2010-14 | NA |  |
|  | - Do you have any physical or mental health conditions or illnesses lasting or expecting to last 12 months or more? | Jan-Dec 2014-21 | NA |  |
|  | - Does your condition or illness reduce your ability to carry out day-to-day activities?  - For how long has your ability to carry-out day-to-day activities been reduced? | Jan-Dec 2015-21 | NA |  |
| British Election Study | - Are your day-to-day activities limited because of a health problem or disability which has lasted, or is expected to last, at least 12 months? | 2014-23 | NA | 1 |
| British Social Attitudes Survey | - Do you have a long-standing physical or mental health condition or disability? By long-standing, I mean anything that has lasted at least 12 months or that is likely to last at least 12 months? - Does this condition or disability have a substantial adverse effect on your ability to carry out normal day-to-day activities? | 2010-20 | NA | 2 |
| Childcare and Early Years Provision | - Do you have a long-standing physical or mental impairment, illness, or disability that you expect will last for at least 12 months? - Does this long standing illness or disability affect the kind of paid work that you might do? - [Do / Does] [you / (partner’s name)] have any illness or disability that you expect will last for at least 12 months? | 2010-13 | NA | 5 |
|  | - Does [child’s name] have any long-standing physical or mental impairment, illness, or disability? By ‘longstanding’ I mean anything that has affected him/her over a period of at least 12 months or that is likely to affect him/her over a period of at least 12 months?  - Does [child’s name]’s long term illness, impairment or disability disrupt their current daily life? | 2014-15; 2017; 2019 | NA |  |
| Citizenship Survey | - Do you have any long-standing illness, disability or infirmity? By long-standing I mean anything that has troubled you over a period of time or that is likely to affect you over a period of time? - Does the illness or disability limit your activities in any way? | 2010-11 | NA | 2 |
| Community Life Survey | - Do you have any physical or mental health conditions or illnesses lasting or expected to last for 12 months or more? - Does your condition or illness/do any of your conditions or illnesses] reduce your ability to carry out day-to-day activities? | 2012-21 | NA | 3 |
|  | **-** Do any of these conditions or illnesses affect you in any of the areas shown on this card? | 2012 | NA |  |
| Continuous Household Survey | - Do you have any long-standing illness, disability or infirmity? By long-standing I mean anything that has troubled you over a period of time or that is likely to affect you over a period of time. **-** Would you mind telling me what this illness or infirmity is?  - Does this illness or disability limit your activities in any way? - Do you have responsibility for the care of a person with a disability or a dependant elderly person? | 2010-11 | NA | 9 |
|  | - Do you have any physical or mental health conditions or illnesses lasting or expecting to last for 12 months or more?  - Does your condition or illness/ do any of your conditions or illnesses reduce your ability to carry out day to day activities? - Do you have responsibility for the care of a person with a disability or a dependant elderly person? | 2012-17 | NA |  |
|  | - Does your condition(s) or illness(es) affect you in any of the following areas? | 2012-15 | NA |  |
|  | **-** For how long has your ability to carry-out day-to-day activities been reduced? | 2012-16 | NA |  |
| Crime Survey for England and Wales | ADULT [16+] - Do you have any of the following long-standing physical or mental health conditions or disabilities that have lasted or are expected to last 12 months or more? - [Does/do] your health condition[s] or [disability/disabilities] mean that your day today activities are limited? for longer than three months. | 2011 | NA | 7 |
|  | ADULT [16+] - Do you have any physical or mental health conditions or illnesses lasting or expected to last for 12 months or more? - Do any of these conditions or illnesses affect you in any of the areas shown on this card?  - [Does your condition or illness/do any of your conditions or illnesses] reduce your ability to carry-out day-to-day activities? | 2012-2020 | NA |  |
|  | CHILD [0-15] - Do you have any long term illness or disability? By long term I mean anything that has affected you for longer than three months or that is likely to affect you for longer than three months.  - Does this illness or disability limit your activities in any way? | 2011-2020 | NA |  |
| English Housing Survey | - Do you have any long-standing illness, disability or infirmity - by long-standing I mean anything that has troubled you over a period of time or that is likely to affect you over a period of time? **-** Does this illness or disability (Do any of these illnesses or disabilities) limit ^names activities in any way?  - What type of illness or disability do you/does [name] have? - Are you/they registered as a disabled person (or as visually impaired) with the local council/ social services? | 2010-11 | NA | 10 |
|  | - This question asks you about any health conditions, illnesses or impairments you may have. Do you have physical or mental health conditions or illnesses lasting or expected to last for 12 months or more? - This question asks about whether your health condition or illness currently affects your ability to carry out normal day-to-day activities, either a lot or a little or not at all. In answering this question, you should consider whether you are affected whilst receiving any treatment or medication for your condition or illness and/or using any devices such as a hearing aid, for example. Does your condition or illness (do any of your conditions or illnesses) reduce your ability to carry out day-to-day activities ... | 2012-20 | NA |  |
|  | - What type of illness or disability do you/does [name] have? | 2012 | NA |  |
|  | - Do any of these conditions or illnesses affect you in any of the following areas? | 2013-20 | NA |  |
|  | - For how long has your ability to carry out day-to-day activities been reduced... - Are you/they registered as a disabled person (or as visually impaired) with the local council/ social services? | 2012-18 | NA |  |
| English Longitudinal Study of Ageing | - [^Do you / Does [^name]] have any long-standing illness, disability or infirmity? By long-standing I mean anything that has troubled [^you / [^name]] over a period of time, or that is likely to affect [^you / [^name]] over a period of time.  - (Does this / Do these) illness(es) or disability(ies) limit [^your / [^name’s]] activities in any way? - You mentioned earlier that you have a health problem or disability that limits the kind or amount of paid work you can do, does this health problem or disability limit the kind or amount of work you can do in your current job? - Is this a health problem or disability that you expect to last less than three months? | 2010-19 | 5-9 | 6 |
|  | - Do you have any health problem that limits the kind or amount of paid work you could do, should you want to? | 2010-15 | 5-7 |  |
|  | - Do you have any health problem or disability that limits the kind or amount of paid work you could do, should you want to? | 2016-19 | 8-9 |  |
| Family Resources Survey | - Do you have any long-standing illness, disability or infirmity? By 'long-standing' I mean anything that has troubled you over a period of at least 12 months or that is likely to affect you over a period of at least 12 months. - Does this physical or mental illness or disability (Do any of these physical or mental illnesses or disabilities) limit your activities in any way? - Does this/Do these health problem(s) or disability(ies) mean that you have substantial difficulties with any of these areas of your life? - Can I just check, do you receive medication or treatment without which your health problems (when taken together), would substantially affect your life in the areas we have been discussing? | 2010-11 | NA | 9 |
|  | - Do you have any physical or mental health conditions or illnesses lasting or expected to last for 12 months or more?  - Do any of these conditions or illnesses affect you in any of the following areas? - Does your condition or illness/do any of your conditions or illnesses reduce your ability to carry-out day-to-day activities? - For how long has your ability to carry-out day-to-day activities been reduced?  - Can I just check, do you receive medication or treatment without which your conditions or illnesses (when taken together), would reduce your ability to carry out day to day activities? | 2012-21 | NA |  |
| Health Survey for England | - Do you have any long-standing illness, disability or infirmity? By long-standing I mean anything that has troubled you over a period of time, or that is likely to affect you over a period of time? **-** Does this illness or disability/do any of these illnesses or disabilities limit your activities in any way? | 2010-11 | NA | 2 |
| Households Below Average Income^**^ | - Individuals who reported substantial difficulties across eight areas of life | 2011 | NA | 2 |
|  | - Individuals who reported any physical or mental health condition(s) or illness(es) that last or are expected to last 12 months or more, and which limit their ability to carry out day-today activities a little, or a lot. | 2012-22 | NA |  |
| Life Opportunities Survey | - Do you have any long-standing physical or mental impairment, illness or disability? By 'long-standing' I mean anything that has affected you over a period of at least 12 months or that is likely to affect you over a period of at least 12 months.  - Does this/Do these health problem(s) or disability(ies) mean that you have substantial difficulties with any of these areas of your life? | 2009-12 | 1-2 | 8 |
|  | - Do you have any physical or mental health conditions or illnesses lasting or expected to last for 12 months or more? **-** Does your condition or illness/do any of your conditions or illnesses reduce your ability to carry-out day-to-day activities? **-** For how long has your ability to carry-out day-to-day activities been reduced? - Can I just check, do you receive medication or treatment without which your health problems (when taken together), would substantially affect your life in the areas we have been discussing? | 2012-14 | 3 |  |
|  | - Do you have any long-standing illness, disability or infirmity - by long-standing I mean anything that has troubled you over a period of time or that is likely to affect you over a period of time?  - Does this illness or disability (Do any of these illnesses or disabilities) limit your activities in any way? | 2009-11 | 1 |  |
| National Child Development Study | - Any physical/mental health conditions lasting or expected to last 12 months - Whether illnesses/conditions reduce ability to carry out day to day activities | 2013 | NA | 2 |
| National Survey for Wales | - Do you have any physical or mental illnesses lasting or expected to last for 12 months or more? | 2012-15, 2016-19, 2021 | NA | 6 |
|  | - Does your condition or illness reduce your ability to carry-out day-to-day activities? | 2012-15, 2016, 2021 | NA |  |
|  | - Do you have any disability or other long standing health problem that makes it or would make it difficult or impossible for you to ride a bicycle?  - Do you have any disability or other long standing health problem that makes it or would make it difficult or impossible for you to walk for more than 5 minutes? | 2013 | NA |  |
|  | - Do this condition limit your ability to carry-out normal day-to-day activities in any way? [Please consider whether you are affected while receiving any treatment, taking medication, or using any devices, such as a hearing aid.] | 2017, 2018 | NA |  |
|  | - Can I just check, do you consider yourself disabled? | 2019 | NA |  |
| National Survey of Sexual Attitudes and Lifestyles | - Do you have any long-standing illness, disability or infirmity? By long-standing I mean anything that has troubled you over a period of time, or that is likely to affect you over a period of time? - Does this limit your activities in any way? | 2010-12 | NA | 4 |
|  | - Do you have any physical or mental health conditions or illnesses lasting or expected to last for 12 months or more? - Do any of your conditions or illnesses reduce your ability to carry out day-to-day activities? | 2020-21 | NA |  |
| National Travel Survey | - Do you have any disability or other long standing health problem that makes it difficult for you to do any of the following… **-** And do you have any other disability of long standing health problem that limits your activities in any other way? By ‘long standing’ I mean anything that has troubled you over a period of at least 12 months or that is likely to affect you over a period of at least 12 months. | 2010-19 | NA | 2 |
| Next Steps (prev. Longitudinal Study of Young People in England) | - Do you have any longstanding illness, disability, or infirmity? By longstanding I mean anything that has troubled you over a period of time or that is likely to affect you over a period of time? **-** Does this illness or disability (Do any of these illnesses or disabilities) limit your activities in any way? | NA | 1 | 5 |
|  | **-** Do you currently have any long-standing physical or mental impairment, illness or disability? 'Long-standing' means anything that has affected you over a period of at least 12 months or that is likely to affect you over a period of at least 12 months. **-** Does this/Do these health problem(s) or disability(ies) mean that you have substantial difficulties with any of these areas of your life?  **-** Do you receive medication or treatment without which your health problems (when taken together), would substantially affect your life in the areas previously mentioned? | NA | 4-7 |  |
| Northern Ireland Life and Times Survey | - Do you have a long-standing illness, disability or infirmity? By long-standing I mean anything that has troubled you over a period of time or that is likely to affect you over a period of time? **-** Does this illness or disability limit your activities in any way? **-** May I check, is there anyone living with you who is sick, disabled or elderly whom you look after or give special help to (for example, a sick, disabled or elderly relative, wife, husband, partner, child, friend)? **-** What about people not living with you, do you provide some regular service or help for any sick, disabled or elderly relative, friend or neighbour not living with you? | 2010-21 | NA | 4 |
| Opinions and Lifestyle Survey | - Have you any long-standing illness, disability or infirmity? **-** Does this Illness / disability limit any of your activities? | 2010-13 | NA | 4 |
|  | **-** Do you have any physical or mental health conditions or illnesses lasting or expected to last for 12 months or more? **-** Do any of your conditions or illnesses reduce your ability to carry out day to day activities? | 2016-17 | NA |  |
| Scottish Crime and Justice Survey | - Do you have any of the following conditions which have lasted, or are expected to last, at least 12 months? **-** Are your day-to-day activities limited because of a health problem or disability which has lasted, or is expected to last, at least 12 months? Please include problems related to old age. | 2010-11 | NA | 4 |
|  | **-** Do you / Does anyone in the household look after, or give any regular help or support to family members, friends, neighbours or others because of either long-term physical or mental ill-health or disability; or problems related to old age? | 2012 | NA |  |
|  | - Apart from anything you do as part of paid employment, do you look after, or give any regular help or support to family members, friends, neighbours or others because of either long-term physical or mental ill-health or disability; or problems related to old age? | 2013-20 | NA |  |
| Scottish Health Survey | - Do you have a long-standing physical or mental condition or disability that has troubled you for at least 12 months, or that is likely to affect you for at least 12 months?  **-** What (else) is the matter with you? **-** Do Does (name of condition) limit your activities in any way? | 2010-11 | NA | 6 |
|  | **-** Do you have a physical or mental condition or illness lasting, or expected to last 12 months or more?  **-** What (else) is the matter with you? **-** Does (name of condition) limit your activities in any way? | 2012-20 | NA |  |
| Scottish Household Survey | **-** Could I just check, do you have any long-standing illness, health problem or disability that limits your daily activities or the kind of work that you can do? By disability as opposed to ill-health, I mean a physical or mental impairment, which has a substantial and long-term adverse effect on your ability to carry out normal day-to-day activities. **-** Could you tell me whether each of the people in the household has any long-standing illness, health problem or disability that limits your/their daily activity or the kind of work that you/they can do? By disability as opposed to ill-health, I mean a physical or mental impairment, which has a substantial and long-term adverse effect on their ability to carry out normal day to day activities.  - Which of the conditions listed on this card best describes the ill-health or disability that (name) has? - How long has (person) been disabled or had a long-term illness? | 2010-19 | NA | 4 |
| Scottish Social Attitudes Survey | - Do you have any long-term illness, health problems or disability. By long-term we mean that it can be expected to last for a year or more? | 2010-16 | NA | 1 |
| UK Census | - Are your day-to-day activities limited because of a health problem or disability which has lasted, or is expected to last, at least 12 months? | 2011 | NA | 3 |
|  | **-** Do you have any physical or mental health conditions or illnesses lasting or expected to last for 12 months or more? - Do any of your conditions or illnesses reduce your ability to carry-out day-to-day activities? | 2021 | NA |  |
| Understanding Society | - Do you have any long-standing physical or mental impairment, illness or disability? By 'long-standing' I mean anything that has troubled you over a period of at least 12 months or that is likely to trouble you over a period of at least 12 months. **-** Does this/Do these health problem(s) or disability(ies) mean that you have substantial difficulties with any of these areas of your life? | 2009-21 | 1-12 | 2 |
| Wealth and Assets Survey | - Do you have any long-standing illness, disability or infirmity? By long-standing I mean anything that has troubled you over a period of time or that is likely to affect you over a period of time? - Does this illness or disability (Do these illnesses or disabilities) limit your activities in any way? - Does this health problem(s) or disability(ies) mean that you have substantial difficulties with any of these areas of your life? | 2010-18 | 3-6 | 3 |
| Welsh Health Survey | - Do you have any long-term illness, health problem or disability which limits your daily activities or the work you can do? (Include problems which are due to old age) - Do you look after, or give any help or support to family members, friends, neighbours or others because of long-term physical or mental ill-health or disability, or problems related to old age? - Do you have any long-standing illness, disability or health problem? That is, anything you have had for some time. **-** Do any of these long-term illnesses, health problems or disabilities limit your daily activities? | 2010-15 | NA | 4 |
| Workplace Employment Relations Survey | - …have a long-term disability that affects the amount or type of work they can do? A ‘long-term disability’ is an illness, health problem or disability that can be expected to last for more than one year. | 2011 | NA | 1 |
| 1970 British Cohort Study | - Any physical/mental health conditions lasting or expected to last 12 months **-** Whether illnesses/conditions reduce ability to carry out day to day activities | 2016-18 | NA | 2 |

^†^Source: UK Data Service: <https://ukdataservice.ac.uk/find-data/>. ^*^It excludes disability benefits specific questions. ^**^No questionnaire available (based on disability guidance). NA: Not Available.

Notes: The following surveys were excluded: Continuous Recording of Social Housing Lettings and Sales (no disability questions per se other than disability-related adaptations); Effects of Taxes and Benefits on Household Income (no disability questions other than benefits); European Working Conditions Survey (no disability questions); General Lifestyle Survey (no access with End User Licence from 2010 onwards); Growing up in Scotland (no access with End User Licence); International Passenger Survey (no disability questions); Living Costs and Food Survey (no disability questions other than benefits); Millenium Cohort Study (only scattered disability benefits questions in the parent module); National Awareness and Early Diagnosis Initiative: Cancer Awareness Measures (no disability questions); Psychiatric Morbidity Surveys (no access with End User Licence from 2010 onwards); Survey of Personal Incomes (no access with End User Licence); Youth Cohort Study (no data from 2010 onwards).
